# Real-Time navigation calibration for pedicle screw placement in neurofibromatosis Type 1–associated scoliosis: An anatomical variability–stratified accuracy study

**DOI:** 10.1007/s00264-026-06907-y

**Published:** 2026-06-25

**Authors:** Guojun Li, Rongjin Feng, Huiqi Qian, Zhongsen Meng, Zhengyi Fu, Tao Chen, Wangqiang Wen, Kaizhong Zhu

**Affiliations:** Key Laboratory of Emergency and Trauma of Ministry of Education, Department of Spinal Surgery, The First Affiliated Hospital, Hainan Medical University, Haikou, China

**Keywords:** Neurofibromatosis type 1, Scoliosis, Navigation calibration, Pedicle classification

## Abstract

**Objective:**

To evaluate whether real-time intraoperative navigation calibration improves pedicle screw placement accuracy in patients with neurofibromatosis type 1–associated scoliosis.

**Methods:**

This retrospective study included 63 patients with NF1-associated scoliosis who underwent posterior spinal osteotomy, deformity correction, and instrumented fixation between January 2023 and June 2025. Patients were assigned to a conventional navigation group (n = 30) or a real-time navigation calibration group (n = 33). Pedicle morphology was classified according to Yang's classification, and screw placement accuracy was assessed on postoperative CT using the Rao grading system. Grade 0 indicated no cortical breach; Grades 1, 2, and 3 indicated breaches of < 2 mm, 2–4 mm, and > 4 mm, respectively.

**Results:**

A total of 430 pedicles were analyzed, including 230 in the conventional navigation group and 200 in the navigation calibration group. The distribution of pedicle morphology did not differ significantly between the groups. A total of 1,228 pedicle screws were inserted (584 in the conventional navigation group and 644 in the navigation calibration group). The navigation calibration group achieved significantly higher Grade 0 placement rates than the conventional navigation group in Type A, B, and C pedicles on the concave side and in Type A and B pedicles on the convex side. The overall cortical breach rate was also lower in the navigation calibration group. No significant between-group differences were observed in operative time, intraoperative blood loss, or perioperative complications.

**Conclusion:**

Real-time intraoperative navigation calibration improved pedicle screw placement accuracy and reduced cortical breaches in NF1-associated scoliosis, particularly in dysplastic pedicles and on the concave side of the deformity. This technique may serve as a practical adjunct to conventional optical navigation in complex spinal deformity surgery.

## Introduction

Neurofibromatosis Type 1 (NF-1) is an autosomal dominant genetic disorder, with scoliosis being its most common skeletal manifestation, reported in up to 60% of patients [1–3]. Affected individuals often present with early-onset spinal deformities, which can be classified as dystrophic or non-dystrophic based on radiographic characteristics [4]. Dystrophic NF-1–associated scoliosis typically progresses rapidly and responds poorly to conservative management, making surgical intervention—such as growing rod techniques or spinal fusion—the primary treatment approach [5–7].

Patients with NF-1–associated scoliosis (NF-1-S) frequently exhibit pathological features including vertebral scalloping, pedicle dysplasia, and osteopenia. Compared with adolescent idiopathic scoliosis, NF1-associated scoliosis often shows greater anatomical distortion, substantially increasing the technical difficulty and risk associated with pedicle screw placement [8, 9]. Accurate screw insertion is crucial for surgical success. Given the proximity of the pedicles to critical structures such as the spinal cord, nerve roots, and major blood vessels, misplacement can lead to severe complications, including neurological deficits or vascular injury, potentially resulting in paralysis or life-threatening outcomes [10, 11]. Achieving precise and safe pedicle screw placement is therefore essential for both surgical safety and correction efficacy.

Conventional freehand techniques primarily rely on the surgeon’s experience with anatomical landmarks, supplemented by intraoperative fluoroscopy. Nevertheless, reported screw misplacement rates remain relatively high, ranging from 8.3% to 50.6% [12–14]. The advent of computer-assisted navigation has significantly improved pedicle screw placement accuracy, with reported misplacement rates as low as 0.3% [15–17]. Integration of three-dimensional navigation with intraoperative imaging further enhances procedural safety and stability, markedly reducing revision rates [18–20]. However, optical navigation systems are still limited by factors such as line-of-sight obstruction and image drift, which can compromise localization accuracy and increase procedural risk [21, 22].

Conventional optical navigation improves the three-dimensional visualization of pedicle trajectories, but its accuracy may be compromised by reference-frame instability, line-of-sight interruption, vertebral micromotion, and image drift[23, 24]. These limitations are particularly relevant in NF1-associated scoliosis, in which dysplastic pedicles and severe vertebral rotation leave little tolerance for navigation error. Therefore, this study aimed to compare pedicle screw placement accuracy between conventional navigation and real-time intraoperative navigation calibration in patients with NF1-associated scoliosis.

## Materials and Methods

### Patient Characteristics

The navigation calibration group included 33 patients, whereas the conventional navigation group included 30 patients. Baseline demographic and radiographic variables are summarized in Table 1, with no significant between-group differences.

**Table 1:** Demographics of the Navigation Calibration Group and the Conventional Navigation Group

	Navigation Calibration Group(n = 33)	Conventional Navigation Group(n = 30)	P
Gender(Male/Female)	14/19	15/15	0.474
Age at surgery (years)	12.25 ± 3.72	13.90 ± 4.66	0.232
Height(m)	1.48 ± 0.16	1.53 ± 0.20	0.304
Weight (kg)	43.67 ± 8.81	47.36 ± 11.49	0.242
BMI	19.81 ± 2.56	19.76 ± 2.49	0.957
Risser sign	3.77 ± 0.95	3.82 ± 1.07	0.863
Preoperative main thoracic curve Cobb angle°	89.94 ± 11.77	91.97 ± 12.29	0.589
Postoperative main thoracic curve Cobb angle°	15.92 ± 4.89	15.29 ± 5.43	0.695
Main thoracic curve correction rate(%)	81.65 ± 6.33	83.14 ± 6.29	0.453
Number of screws placed per case	19.52 ± 0.49	19.48 ± 0.56	0.128

**Inclusion criteria**

1. Diagnosis meeting the clinical criteria for NF-1;
2. Surgical indication independently confirmed by two senior spine surgeons;
3. All procedures performed by the same experienced chief surgeon using a posterior osteotomy, deformity correction, and internal fixation approach, with perioperative management provided by a consistent clinical team;
4. Availability of complete preoperative data, postoperative imaging, and follow-up records [25].

**Exclusion criteria:**

1. Scoliosis not attributable to NF-1;
2. Presence of severe osteoporosis, infection, tumor, or other contraindications to posterior corrective surgery with internal fixation;
3. Incomplete medical or follow-up records;
4. Contraindications to surgery or anesthesia;
5. History of prior spinal surgery.

### Surgical procedures

All procedures were performed by the same chief surgeon with over 20 years of experience in spine surgery, assisted by a consistent surgical team.

#### Anaesthesia, positioning, and neuromonitoring

All patients underwent combined intravenous–inhalational general anaesthesia and were positioned prone on a carbon fibre operating table. Standard sterile preparation and draping were performed. After induction of anaesthesia, intraoperative neuromonitoring was routinely established, including somatosensory evoked potentials (SSEPs) and motor evoked potentials (MEPs). Alarm criteria were defined as a > 50% decrease in MEP amplitude or a > 10% increase in SSEP latency. Spinal cord function was continuously monitored throughout the procedure [26].

#### Surgical exposure and reference frame placement

A standard posterior midline longitudinal incision was made, followed by subperiosteal dissection to expose the spinous processes, laminae, and facet joints. A navigation reference frame was rigidly fixed to one iliac crest. Intraoperative three-dimensional imaging of the target region was obtained using a Siemens ISO-C 3D C-arm system, and the imaging data were imported into the Brainlab navigation workstation for automatic registration.

#### Pedicle screw placement

Conventional navigation group:

Under real-time guidance using a navigation probe, the optimal entry point and the transverse and sagittal trajectories were determined. A pilot hole was created using a high-speed burr, followed by pedicle cannulation with a pedicle probe. A ball-tipped probe was used to confirm the integrity of the pedicle walls before screw insertion. Care was taken to avoid displacement of the reference frame and interference with the navigation system.

Navigation calibration group:

In the navigation calibration group, an 18-gauge sterile calibration pin was inserted into the spinous process of the target vertebra after exposure. The position of the pin was verified using the navigation probe and compared with the corresponding point on the navigation images. When both the translational deviation was ≤ 2 mm and the angular deviation was ≤ 5°, navigation accuracy was considered acceptable, and minor trajectory corrections were incorporated before screw insertion. If either threshold was exceeded, the reference frame, optical tracking pathway, and instrument registration were rechecked; repeat three-dimensional imaging was performed when necessary. Screw insertion was then performed using the same workflow as in the conventional navigation group.

#### Deformity correction, fusion, and wound closure

After all pedicle screws were inserted, pre-contoured 5.5-mm titanium rods were applied. Three-dimensional deformity correction was achieved using rod rotation, compression, distraction, and direct vertebral derotation techniques. Following thorough decortication of the laminae, autologous iliac bone graft or allograft bone was placed onto the prepared fusion bed. A drainage tube was inserted, and the incision was closed in layers.

#### Postoperative management

Postoperative management included prophylactic antibiotics, analgesia, neurological monitoring, and standardized rehabilitation.

#### Outcome measures

Based on the pedicle classification system proposed by Yang et al. [27], the transverse width at the narrowest point of each pedicle from T1 to L5 was measured on axial CT images. NF-1–associated pedicles were categorized as follows: Type A (cancellous channel > 4 mm), Type B (cancellous channel 2–4 mm), Type C (cancellous channel < 2 mm but with an intact cortical channel ≥ 2 mm), Type D (cortical channel < 2 mm), and Type E (absence of the pedicle). Type A was defined as a normal pedicle, whereas Types B, C, D, and E were defined as abnormal pedicles because of progressive narrowing, cortical deficiency, or absence of the pedicle channel, indicating insufficient width for safe screw placement.

Postoperative CT scans were used to evaluate the accuracy of pedicle screw placement. Screw accuracy was assessed by measuring the deviation between the planned and actual screw positions, and determining the presence of pedicle wall breach. All screw positions were independently evaluated by two spine surgeons who were not involved in the surgical procedures. Discrepancies were resolved through consensus or adjudication by a third senior surgeon. Screw placement was graded according to the classification system described by Rao: Grade 0 (no cortical breach), Grade 1 (breach < 2 mm), Grade 2 (breach 2–4 mm), and Grade 3 (breach > 4 mm).Perioperative parameters were recorded for both groups, including operative time, intraoperative blood loss, and complications,.

### Statistical analysis

All statistical analyses were performed using SPSS version 26.0 (IBM Corp., Armonk, NY, USA). Normality was assessed using the Shapiro–Wilk test. Continuous variables are reported as mean ± standard deviation or median (interquartile range), as appropriate. Categorical variables are reported as counts and percentages. Between-group comparisons were performed using the independent-samples t test, Mann–Whitney U test, chi-square test, or Fisher exact test, as appropriate. Ordinal screw breach grades were compared using the Mann–Whitney U test. Two-sided P values < 0.05 were considered statistically significant. Subgroup analyses stratified by pedicle type and deformity side were performed as exploratory analyses to identify potential anatomical patterns of screw placement accuracy. No formal adjustment for multiple comparisons was applied; therefore, subgroup-level P values were interpreted cautiously and were not regarded as confirmatory evidence.

## Results

### General characteristics

**Fig. 1:**
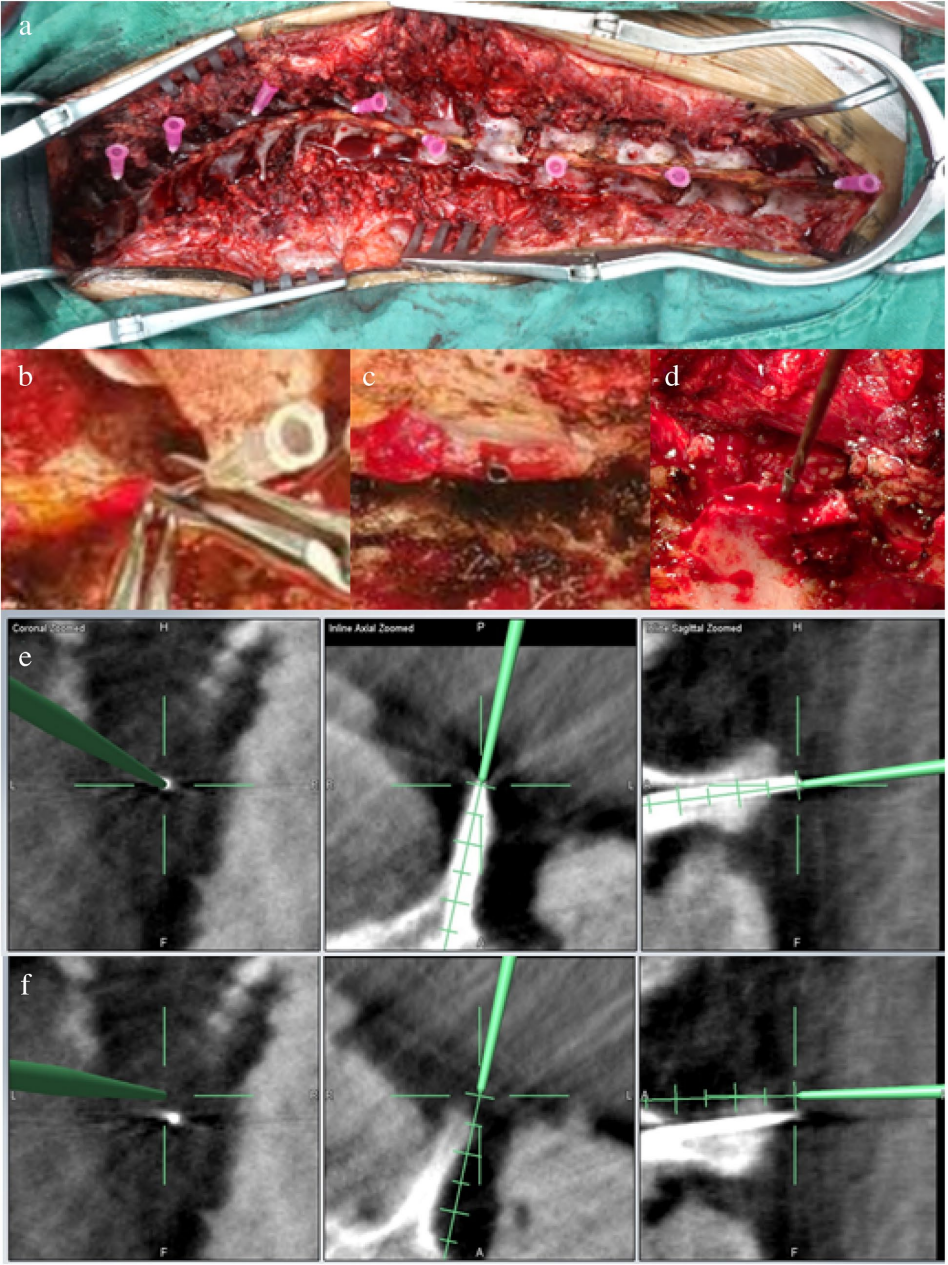
Schematic illustration of computer-assisted navigation image calibration and pedicle screw placement. a. Insertion of a syringe needle into the spinous process after complete exposure of the lamina and spinous process. b–c. Removal of the syringe tip to create a calibration reference.d. Placement of the tracker on the guide needle for positional verification.e. Alignment of the navigation workstation image with the calibration reference in both sagittal and axial planes.f. Illustration of the navigation image showing an upward displacement of 2.1 mm in the axial plane

**Fig. 2:**
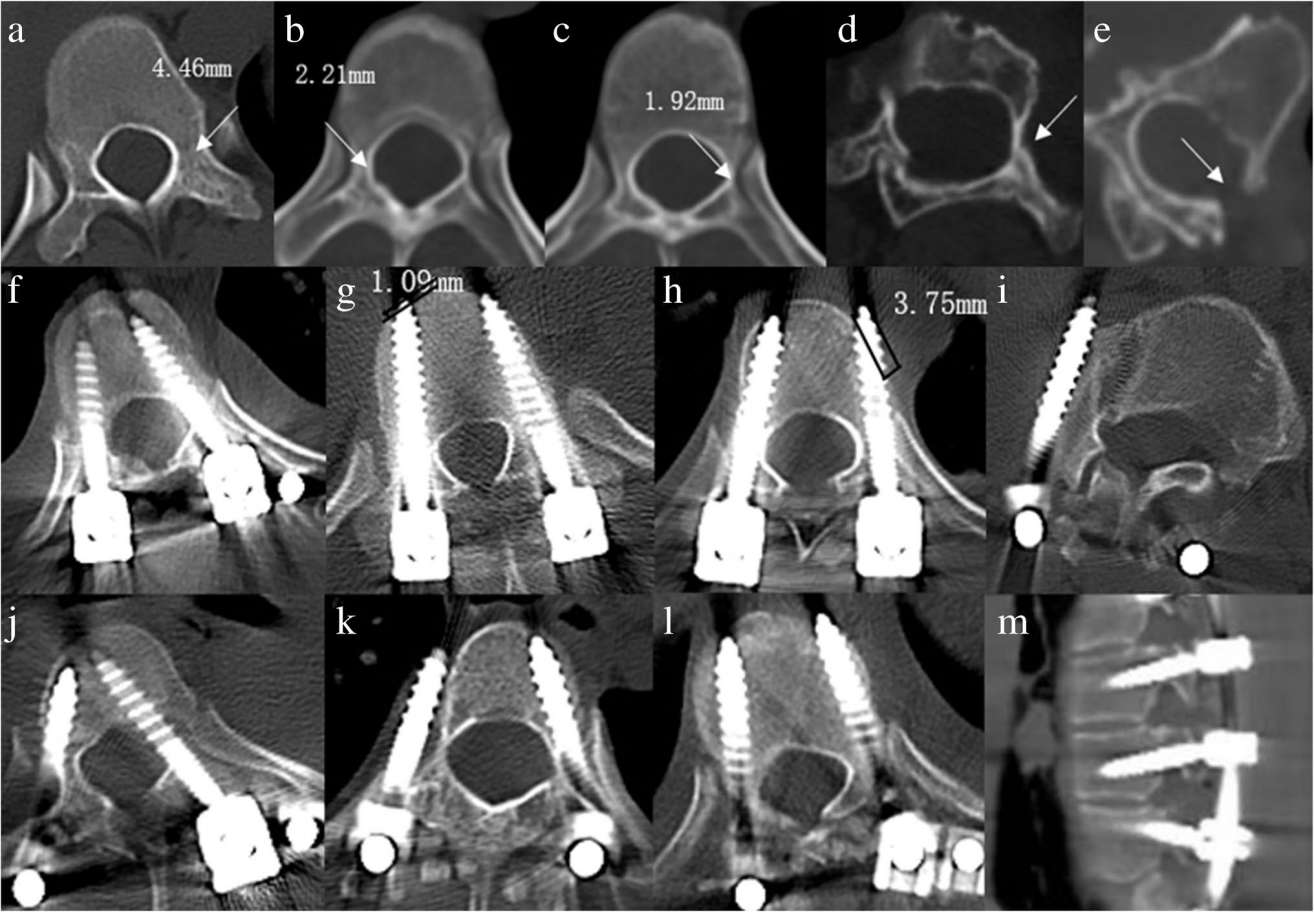
Pedicle classification, pedicle screw breach grading, and breach direction. a:​ Type A (Cancellous bone channel > 4 mm);b:​ Type B (Cancellous bone channel 2–4 mm);c:​ Type C (Cancellous bone channel < 2 mm, but cortical bone channel intact and ≥ 2 mm);d:​ Type D (Cortical bone channel < 2 mm);e:​ Type E (Pedicle absent);f:​ Grade 0 (No cortical breach);g:​ Grade 1 (Breach < 2 mm);h:​ Grade 2 (Breach 2–4 mm);i:​ Grade 3 (Breach > 4 mm);j:​ Medial wall breach; k:​ Lateral wall breach; l:​ Anterior wall breach; m:​ Neuroforaminal breach

Sixty-three patients met the inclusion criteria. The navigation calibration group included 33 patients and 644 screws, whereas the conventional navigation group included 30 patients and 584 screws. Baseline demographic and radiographic characteristics were comparable between the two groups (all P > 0.05). Both groups demonstrated significant reductions in Cobb angle compared with preoperative values. However, there was no statistically significant difference in correction outcomes between the two groups (P > 0.05). (Figure 1 and 2).

### Anatomical characteristics of pedicles in NF-1–associated scoliosis

**Fig. 3:**
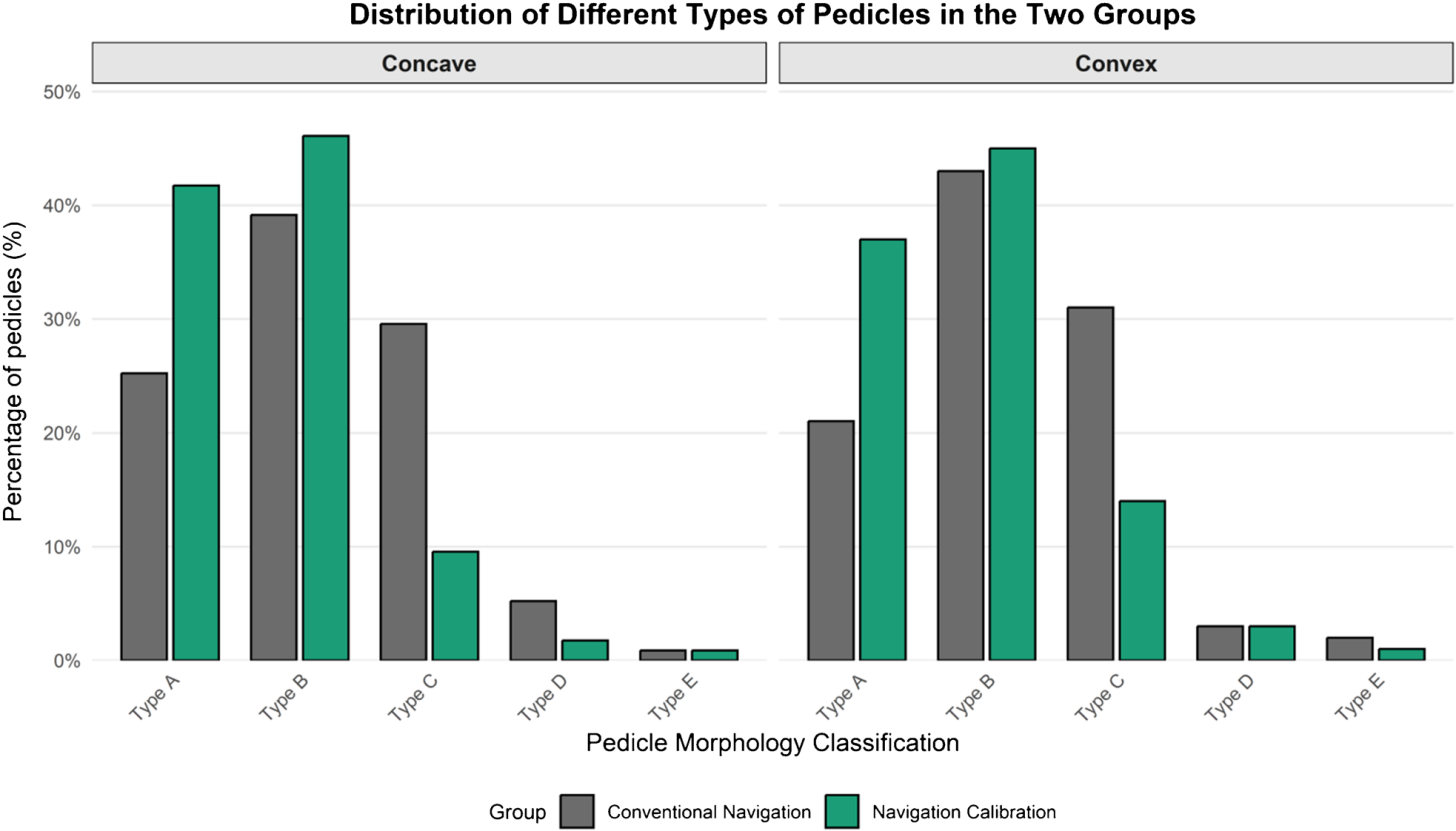
Distribution of pedicle types in the two groups. Comparison of pedicle classification on the concave and convex sides in the apical region revealed no significant difference between the two groups (P > 0.05), indicating good comparability

A total of 430 pedicles in the apical vertebral region were analyzed (Fig. 3): 230 in the conventional navigation group and 200 in the navigation calibration group. Pedicle types were classified according to Yang’s classification system. Conventional navigation group: Type A 77 (33.5%), Type B 98 (42.5%), Type C 45 (19.6%), Type D 8 (3.5%), Type E 2 (0.9%).Navigation calibration group: Type A 58 (29.0%), Type B 88 (44.0%), Type C 45 (22.5%), Type D 6 (3.0%), Type E 3 (1.5%).There was no statistically significant difference in pedicle type distribution between the two groups (P > 0.05).The proportion of normal pedicles (Type A) was significantly lower than that of abnormal pedicles (Types B, C, D, and E) in both groups (conventional navigation: 33.5% vs. 66.5%; navigation calibration: 29.0% vs. 71.0%; P = 0.001). Stratified analysis revealed that on the concave side, abnormal pedicles predominated in both groups (conventional navigation: 74.8% vs. 25.2%; navigation calibration: 79.0% vs. 21.0%; P = 0.001). On the convex side, the navigation calibration group showed a higher proportion of abnormal pedicles (63.0%) compared with normal pedicles (37.0%; P = 0.002).

### Accuracy of pedicle screw placement

**Fig. 4:**
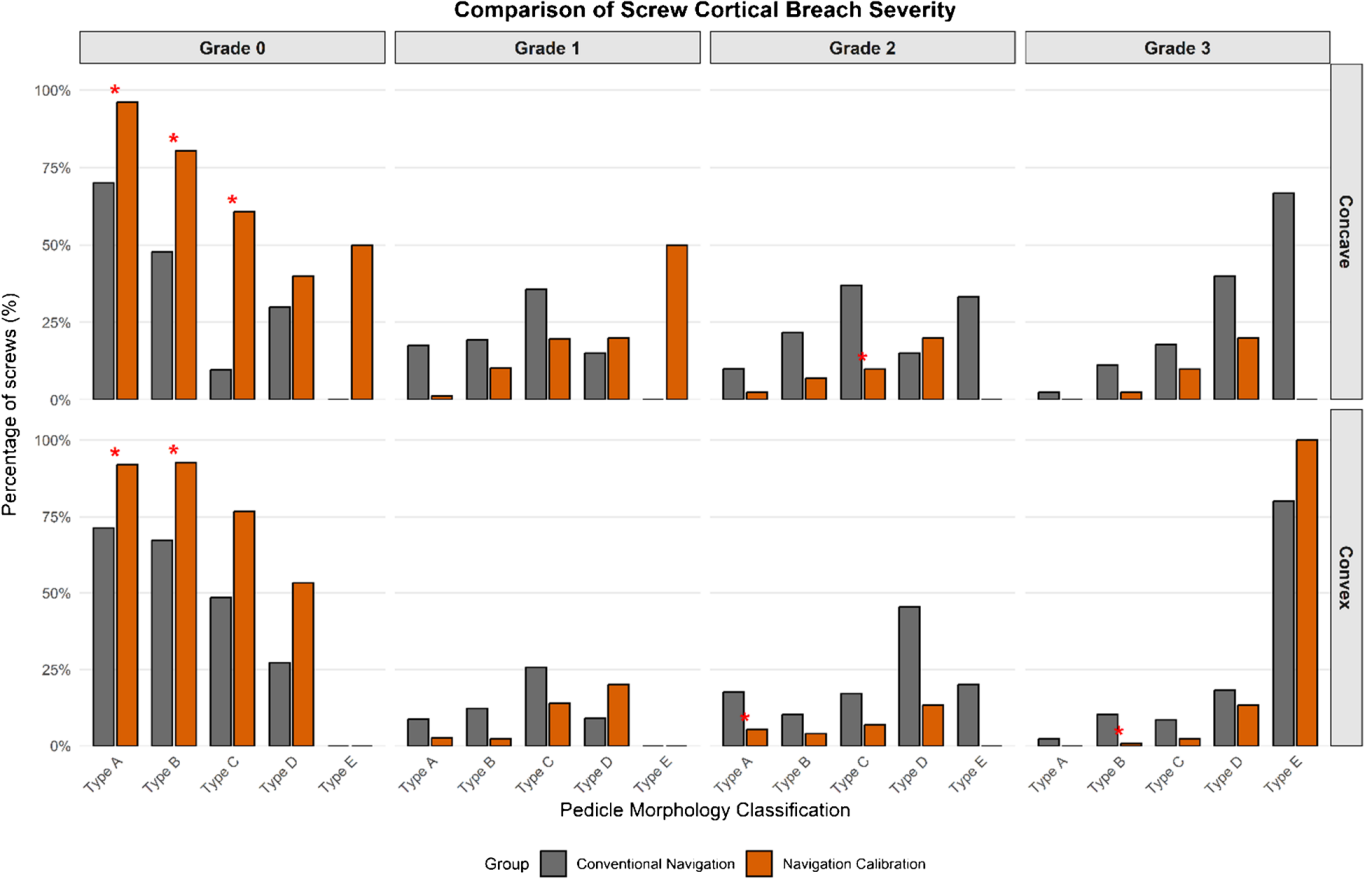
Comparison of cortical breach severity between the two groups. Comparison of breach grades (Grade 0–3) between the two groups across different pedicle types (**A**–**E**) revealed several statistically significant differences.On the concave side, significant differences were observed as follows:Type A: Grade 0 (χ^2^ = 12.438, P = 0.004);Type B: Grade 0 (χ^2^ = 9.550, P = 0.003);Type C: Grade 0 (χ^2^ = 7.541, P = 0.006) and Grade 2 (χ^2^ = 4.816, P = 0.048);On the convex side, significant differences were identified in:Type A: Grade 0 (χ^2^ = 6.564, P = 0.010) and Grade 2 (χ^2^ = 3.645, P = 0.021);Type B: Grade 0 (χ^2^ = 10.096, P = 0.004) and Grade 3 (χ^2^ = 2.427, P = 0.043);P < 0.05 was considered statistically significant

The accuracy of pedicle screw placement is summarized in Fig. 4. On the concave side, the navigation calibration group showed significantly higher Grade 0 screw placement rates than the conventional navigation group in Type A, Type B, and Type C pedicles. On the convex side, Grade 0 placement rates were also significantly higher in Type A and Type B pedicles in the navigation calibration group. In addition, the incidence of higher-grade cortical breaches was reduced in several pedicle subtypes, indicating improved screw placement accuracy after real-time intraoperative calibration.

### Direction of pedicle screw breach

Postoperative imaging assessed the frequency and direction of cortical breaches.Conventional navigation group: 177 breaches on concave side (27.5%), 118 on convex side (18.3%).Navigation calibration group: 63 breaches on concave side (10.8%), 36 on convex side (6.2%).The overall breach rate was significantly lower in the navigation calibration group (P = 0.009). Breach rates were consistently higher on the concave side than the convex side in both groups (P < 0.05).

**Fig. 5:**
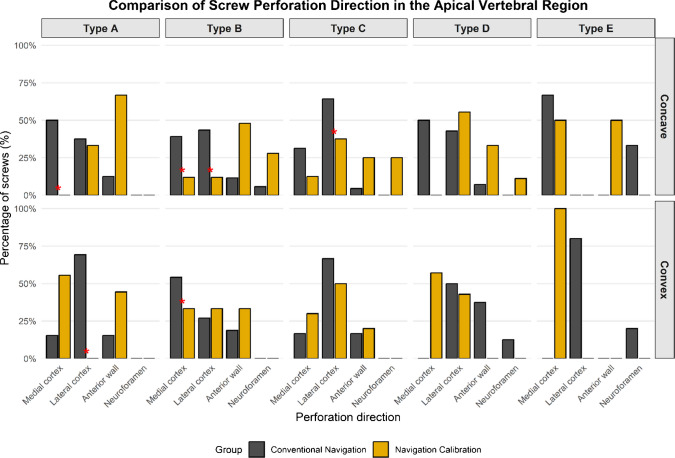
Comparison of Screw Breach direction in the Apical Vertebral Region Between the Two Groups. Comparison of screw breach directions (medial cortex, lateral cortex, anterior wall, and neuroforamen) between the two groups across pedicle types (A–E) revealed several statistically significant differences.On the concave side, significant differences were observed for:Type A: medial cortex (χ^2^ = 4.160, P = 0.041);Type B: medial cortex (χ^2^ = 5.463, P = 0.020) and lateral cortex (χ^2^ = 6.943, P = 0.011);Type C: lateral cortex (χ^2^ = 5.452, P = 0.019);On the convex side, significant differences were found for:Type A: lateral cortex (χ^2^ = 8.152, P = 0.004);Type B: medial cortex (χ^2^ = 4.331, P = 0.037) P < 0.05

Breach pattern by pedicle type: Type A: Medial breach concave: 0.5% vs. 3.7% (P = 0.041); lateral breach convex: 1.5% vs. 6.1% (P = 0.004).Type B: Medial breach concave 4.3% vs. 10.7% (P = 0.020); convex 1.5% vs. 7.5% (P = 0.037); lateral breach concave 0.5% vs. 4.7% (P = 0.011).Type C: Lateral breach concave 1.5% vs. 6.7% (P = 0.019).Type D and E: Limited events; statistical analysis not performed (Fig. 5).

### Clinical outcomes

Perioperative clinical outcomes are summarized in Table 2. There were no significant differences between the navigation calibration and conventional navigation groups in Intraoperative blood loss, operative time, or perioperative complications. No significant differences were observed in delayed wound healing, transient neurological injury, pneumothorax, intercostal neuralgia, or gastrointestinal obstruction. These findings suggest that real-time intraoperative navigation calibration improved screw placement accuracy without increasing additional surgical burden, without increasing operative time, blood loss, or perioperative complications.

**Table 2:** Comparison of Perioperative Clinical Outcomes Between the Two Groups

	Navigation Calibration Group(n = 33)	Conventional Navigation Group(n = 30)	P
Intraoperative blood loss(mL)	652 ± 16.82	624 ± 13.45	0.324
Operative time(min)	354 ± 11.25	320 ± 8.64	0.251
Complications			
Delayed wound healing	2(6%)	1(3.3%)	0.142
Transient neurological injury	1(3%)	0	0.457
Pneumothorax	3(9%)	1(3.3%)	0.063
Intercostal neuralgia	1(3%)	0	0.237
Gastrointestinal obstruction	0	1(3.3%)	0.251

## Discussion

Neurofibromatosis type 1–associated scoliosis (NF-1-S) is a complex spinal deformity characterized by early onset, rapid progression, and marked anatomical variability. In dystrophic scoliosis, vertebral scalloping, pedicle dysplasia, dural ectasia, vertebral rotation, and osteopaenia frequently coexist, resulting in substantial challenges for posterior deformity correction and pedicle screw fixation [23, 24, 28–31]. Because the pedicles are adjacent to the spinal cord, nerve roots, and major vascular structures, inaccurate screw placement may lead to severe neurological or vascular complications [32, 33]. Therefore, improving the safety and accuracy of pedicle screw insertion is a central concern in the surgical treatment of NF-1-S.

The present study demonstrated that abnormal pedicle morphology was highly prevalent in patients with NF-1-S. Type A pedicles accounted for only a minority of apical pedicles, whereas Types B, C, D, and E were more frequently observed, particularly on the concave side. This finding is consistent with previous studies reporting a high incidence of pedicle dysplasia and asymmetric pedicle development in NF-1-related spinal deformity [23, 24, 34]. The predominance of abnormal pedicles on the concave side may be related to vertebral rotation, asymmetric growth, and vertebral body drift, all of which further narrow the pedicle channel and increase the technical difficulty of screw insertion. These anatomical characteristics explain why NF-1-S is associated with a higher risk of screw malposition than idiopathic scoliosis and highlight the importance of precise preoperative assessment and intraoperative guidance.

Computer-assisted navigation has been widely used to improve the accuracy of pedicle screw placement in complex spinal deformity surgery. Compared with the freehand technique, navigation provides real-time three-dimensional visualization of the pedicle trajectory and allows more accurate selection of the entry point, screw direction, and screw diameter. Previous studies have shown that navigation-assisted screw placement can reduce cortical breach rates and improve the proportion of clinically acceptable screws [35, 36]. However, conventional optical navigation is not completely immune to intraoperative errors. Relative motion between vertebrae, loosening of the reference frame, obstruction of the optical tracking pathway, instrument deformation, respiratory motion, and image drift may all compromise navigation accuracy. These errors may be particularly problematic in NF-1-S patients, whose pedicles are often narrow, dysplastic, or partially absent.

To address these limitations, the present study applied a real-time intraoperative navigation calibration technique based on a positioning pin inserted into the spinous process. The main finding was that real-time calibration significantly improved pedicle screw placement accuracy compared with conventional navigation. In particular, the navigation calibration group showed higher Grade 0 screw placement rates in Type A and Type B pedicles on both the concave and convex sides, as well as in Type C pedicles on the concave side. Moreover, the incidence of higher-grade cortical breaches was reduced in several pedicle subtypes. These results suggest that real-time calibration is especially valuable in pedicles with abnormal morphology, where even minor navigation deviation may result in cortical violation.

The potential mechanism underlying this improvement may be related to dynamic verification of navigation accuracy during screw placement. In spinal deformity surgery, the vertebral body, pedicles, and spinous process form an integrated anatomical unit. When vertebral rotation or subtle vertebral displacement occurs during exposure, probing, tapping, or screw insertion, the anatomical entry point and screw trajectory may shift accordingly. Conventional navigation relies on the accuracy of the initial registration and may not fully reflect these dynamic intraoperative changes. By placing a calibration pin into the spinous process, the surgeon can repeatedly verify the consistency between the actual anatomical landmark and the corresponding navigation image. If positional or angular deviation is detected, the navigation trajectory can be adjusted before screw insertion. This process may reduce the risk of screw malposition caused by image drift, vertebral micromotion, or reference frame instability.

Another important finding of this study was that real-time navigation calibration improved screw accuracy without increasing perioperative surgical burden. There were no significant differences between the navigation calibration and conventional navigation groups in intraoperative Intraoperative blood loss, operative time, or perioperative complications. Delayed wound healing, transient neurological injury, pneumothorax, intercostal neuralgia, and gastrointestinal obstruction were comparable between the two groups. These findings indicate that the calibration procedure did not increase operative trauma or complication risk. Given that the technique is simple, repeatable, and can be integrated into the standard navigation workflow, it may represent a practical adjunct for improving screw placement safety in complex NF-1-S surgery.

The clinical significance of this study lies in its focus on a high-risk deformity population with severe anatomical distortion. Although navigation has improved the safety of spinal instrumentation, navigation errors remain a major concern in patients with narrow or dysplastic pedicles. The present results suggest that real-time intraoperative calibration can serve as an additional intraoperative verification strategy beyond conventional navigation registration. This technique may be particularly useful in the apical region, on the concave side, and in Type B or Type C pedicles, where the pedicle channel is narrow but screw placement may still be attempted. By reducing cortical breach rates, real-time calibration may help improve fixation safety and confidence during deformity correction.

Several limitations should be acknowledged. First, this was a retrospective study with a relatively limited sample size because NF-1-S is rare and strict inclusion criteria were applied. The statistical power and generalizability of the results may therefore be limited. Second, all procedures were performed by a single experienced spine surgeon, which improved technical consistency but may have introduced surgeon-related bias. The learning curve associated with navigation calibration may also have influenced the results. Third, this was a single-centre study, and differences in surgical technique, navigation systems, instrumentation, and patient characteristics among institutions may affect the reproducibility of the findings. Fourth, this study mainly focused on radiographic screw accuracy and perioperative outcomes; long-term follow-up data regarding fusion status, implant-related complications, maintenance of correction, implant failure, fusion status, and patient-reported outcomes.

In conclusion, real-time intraoperative navigation calibration significantly improved pedicle screw placement accuracy in patients with NF-1-associated scoliosis, particularly in pedicles with abnormal morphology and on the concave side of the deformity. The technique reduced cortical breach rates without increasing Intraoperative blood loss, operative time, or perioperative complications. Real-time calibration may therefore serve as a useful adjunct to conventional optical navigation and may enhance the safety and precision of pedicle screw placement in complex NF-1-S corrective surgery. Larger multicenter studies with long-term follow-up are needed to further validate these findings and clarify the broader clinical value of this technique.

## Conclusion

This study demonstrates that real-time intraoperative calibration significantly improves the accuracy of pedicle screw placement compared with conventional computer-assisted navigation in patients with NF-1–associated scoliosis. The technique notably reduces the incidence of high-grade cortical breaches, particularly in pedicles with abnormal anatomy. These findings underscore the clinical utility and potential of real-time calibration in enhancing surgical safety and precision in the management of complex spinal deformities.

## Data Availability

No datasets were generated or analysed during the current study.

## References

[1] Heyde CE, Volker A, von der Hoh NH et al (2021) Spinal deformity in neurofibromatosis type 1[J]. ORTHOPADE 50(8):650–656 34236453 10.1007/s00132-021-04130-8

[2] Wang D, Zhang BH, Wen X et al (2024) Clinical features and surgical treatments of scoliosis in neurofibromatosis type 1: a systemic review and meta-analysis. Eur Spine J 33(7):2646–2665. 10.1007/s00586-024-08194-w 38526628 10.1007/s00586-024-08194-w

[3] Huang J, Liu J, Li Y, Yu X, Wang Z, Li Q (2026) The evolving landscape in neurofibromatosis type 1: promise under pressure. Int J Surg 112(2):5224–5227. 10.1097/JS9.0000000000003724 41247802 10.1097/JS9.0000000000003724

[4] Mladenov KV, Stücker R (2024) Recent developments in surgical treatment of spinal deformity in pediatric patients: experience from a single-center series of 42 neurofibromatosis type 1 patients. Cancers (Basel) 16(23):4079. 10.3390/cancers16234079 39682265 10.3390/cancers16234079 PMC11640448

[5] Li H, Zhang W, Yao Z et al (2022) Genotypes and clinical intervention of patients with neurofibromatosis type 1 associated dystrophic scoliosis. Front Pediatr 10:918136 36061378 10.3389/fped.2022.918136 PMC9434403

[6] Yang W, Chen S, Du Y et al (2026) Surgical outcomes in NF-1-associated upper thoracic dystrophic scoliosis: a retrospective analysis. World Neurosurg. 10.1016/j.wneu.2026.124921 41831600 10.1016/j.wneu.2026.124921

[7] Li H, Zhang H, Gao R et al (2026) Surgical treatment for neurofibromatosis type 1-related dystrophic scoliosis in children aged 8 to 11: traditional growing rod or posterior spinal fusion? BMC Surg 26(1):127. 10.1186/s12893-026-03486-y 41545977 10.1186/s12893-026-03486-y PMC12896003

[8] Welborn M, Tambe A, Adeyemi A, Dupuis M, Simoneau D, Brandi ML (2025) Burden of disease and unmet needs associated with scoliosis in neurofibromatosis type 1: a systematic literature review. JBMR Plus 9(8):ziaf072. 10.1093/jbmrpl/ziaf072 40703410 10.1093/jbmrpl/ziaf072 PMC12284884

[9] Sakhrekar R, Shkumat N, Ertl-Wagner B et al (2024) Pedicle screw accuracy placed with assistance of machine vision technology in patients with neuromuscular scoliosis. Spine Deform 12(3):739–746. 10.1007/s43390-024-00830-1 38413472 10.1007/s43390-024-00830-1

[10] Amaral T, Hasan S, Galina J et al (2021) Screw malposition: are there long-term repercussions to malposition of pedicle screws? J Pediatr Orthop 41:S80–S86 34096543 10.1097/BPO.0000000000001828

[11] Sasagawa T (2022) Rate and factors associated with misplacement of percutaneous pedicle screws in the thoracic spine. Spine Surg Relat Res 7(2):155–160. 10.22603/ssrr.2022-0133 37041869 10.22603/ssrr.2022-0133 PMC10083079

[12] Dietz N, Spiessberger A (2024) Consistent anatomical relationships of pedicle, lamina, and superior articulating process in severe idiopathic scoliosis allow for safe freehand pedicle screw placement: a proof-of-concept technical study. J Craniovertebr Junction Spine 15(2):224–229 38957756 10.4103/jcvjs.jcvjs_16_24 PMC11216649

[13] Matur AV, Palmisciano P, Duah HO et al (2023) Robotic and navigated pedicle screws are safer and more accurate than fluoroscopic freehand screws: a systematic review and meta-analysis[J]. Spine Journal 23(2):197–298 10.1016/j.spinee.2022.10.006 36273761

[14] Li C, Wang Z, Li D et al (2023) Safety and accuracy of cannulated pedicle screw placement in scoliosis surgery: a comparison of robotic-navigation, O-arm-based navigation, and freehand techniques. Eur Spine J 32(9):3094–3104. 10.1007/s00586-023-07710-8 37273031 10.1007/s00586-023-07710-8

[15] Ishak B, Younsi A, Wieckhusen C et al (2019) Accuracy and revision rate of intraoperative computed tomography point-to-point navigation for lateral mass and pedicle screw placement: 11-year single-center experience in 1054 patients. Neurosurg Rev 42:895–905 30569212 10.1007/s10143-018-01067-z

[16] Papalia GF, Vadalà G, Russo F et al (2024) Higher accuracy and better clinical outcomes in navigated thoraco-lumbar pedicle screw fixation versus conventional techniques. Spine (Phila Pa 1976) 49(19):1370–1380. 10.1097/BRS.0000000000005105 39049509 10.1097/BRS.0000000000005105 PMC11386964

[17] Oba H, Uehara M, Ikegami S et al (2023) Tips and pitfalls to improve accuracy and reduce radiation exposure in intraoperative CT navigation for pediatric scoliosis: a systematic review. Spine J 23(2):183–196. 10.1016/j.spinee.2022.09.004 36174926 10.1016/j.spinee.2022.09.004

[18] Sundaram PPM, Oh JY, Tan M et al (2021) Accuracy of thoracolumbar pedicle screw insertion based on routine use of intraoperative imaging and navigation. Asian Spine J 15(4):491–497 32951407 10.31616/asj.2020.0068 PMC8377205

[19] Li S, Du J, Huang Y et al (2023) Comparison of surgical efficacy between O-arm combined with CT 3D real-time navigation system and Tinavi robot-assisted treatment of adolescent congenital scoliosis[J]. Am J Transl Res 15(5):3254–3266 37303634 PMC10250986

[20] Khashab MA, Elkhalifa M, Alswat MM, Adas RA (2026) Comparison of the accuracy of marker screw-assisted pedicle screw placement in thoracic and lumbar spine to 3D navigation: a randomized controlled study. Glob Spine J. 10.1177/21925682261433515 10.1177/21925682261433515 PMC12975543 41806365

[21] Rahmathulla G, Nottmeier EW, Pirris SM et al (2014) Intraoperative image-guided spinal navigation: technical pitfalls and their avoidance. Neurosurg Focus 36(3):E3 10.3171/2014.1.FOCUS13516 24580004

[22] Cawley D, Dhokia R, Sales J et al (2020) Ten techniques for improving navigated spinal surgery. Bone Joint J 102-B(3):371–375 32114817 10.1302/0301-620X.102B3.BJJ-2019-1499.R1

[23] Pushpa BT, Rajasekaran S, Anand KSSV et al (2022) Anatomical changes in vertebra in dystrophic scoliosis due to neurofibromatosis and its implications on surgical safety. Spine Deform 10(1):159–167 34309821 10.1007/s43390-021-00392-6

[24] Rajasekaran S, Pushpa BT, Anand KSSV et al (2022) The phenomenon of vertebral body drift in neurofibromatosis and its implications for surgical safety. Eur Spine J 31(6):1343–1348 35362735 10.1007/s00586-022-07160-8

[25] Ly KI, Blakeley JO (2019) The diagnosis and management of neurofibromatosis type 1. Med Clin North Am 103(6):1035–1054 31582003 10.1016/j.mcna.2019.07.004

[26] Kramer S, Ford L, Walsh J (2024) Neuromonitoring changes in spinal deformity surgery. Orthop Clin North Am 55(1):89–99 37980106 10.1016/j.ocl.2023.07.002

[27] Yang N, Luo M, Zhao S et al (2020) Morphological differences between the pedicles in nondystrophic scoliosis secondaryto neurofibromatosis type 1 and those in adolescent idiopathic scoliosis. World Neurosurg 144:9–14 10.1016/j.wneu.2020.06.036 32540291

[28] Fujita R, Oda I, Takeuchi H et al (2022) Accuracy of pedicle screw placement using patient-specific template guide system. J Orthop Sci 27(2):348–354 33640220 10.1016/j.jos.2021.01.007

[29] Moodley M, Lopez KR (2024) Neurofibromatosis type 1-an update. Semin Pediatr Neurol 52:101172 39622609 10.1016/j.spen.2024.101172

[30] Stylianides C, Hadjigavriel G, Theotokis P et al (2025) Epigenetic mechanisms in neurofibromatosis types 1 and 2. Epigenomes 9(3):30. 10.3390/epigenomes9030030 40843672 10.3390/epigenomes9030030 PMC12372142

[31] Kaspiris A, Lianou I, Marouglianis V et al (2025) Tips and pitfalls of surgical techniques for scoliotic deformities in neurofibromatosis type 1. J Clin Med 15(1):104. 10.3390/jcm15010104 41517354 10.3390/jcm15010104 PMC12786608

[32] Li S, Mao S, Du C et al (2021) Assessing the unique characteristics associated with surgical treatment of dystrophic lumbar scoliosis secondary to neurofibromatosis type 1: a single-center experience of more than 10 years. J Neurosurg Spine 34(3):413–423 33254143 10.3171/2020.6.SPINE20898

[33] Neifert S N, Khan H A, Kurlanddb, et al. (2022) Management and surgical outcomes of dystrophic scoliosis in neurofibromatosis type 1: a systematic review[J]. Neurosurg Focus 52(5):E7 10.3171/2022.2.FOCUS21790 35535821

[34] Li Y, Luo M, Wang W et al (2017) A computed tomography-based comparison of abnormal vertebrae pedicles between dystrophic and nondystrophic scoliosis in neurofibromatosis type 1[J]. World Neurosurgery 106:898–904 28735128 10.1016/j.wneu.2017.07.064

[35] Sielatycki JA, Mitchell K, Leung E et al (2022) State of the art review of new technologies in spine deformity surgery–robotics and navigation. Spine Deform 10(1):5–17 34487345 10.1007/s43390-021-00403-6 PMC8741671

[36] Li C, Wang Z, Li D et al (2023) Safety and accuracy of cannulated pedicle screw placement in scoliosis surgery: a comparison of robotic-navigation, O-arm-based navigation, and freehand techniques. Eur Spine J 32:3094–3104 37273031 10.1007/s00586-023-07710-8

